# The Predictive Value of Epicardial Fat Tissue Volume in the Occurrence and Development of Atrial Fibrillation: A Systematic Review and Meta-Analysis

**DOI:** 10.1155/2022/2090309

**Published:** 2022-09-29

**Authors:** Qiankun Fan, Yinge Zhan, Mingqi Zheng, Fangfang Ma, Lishuang Ji, Lei Zhang, Gang Liu

**Affiliations:** ^1^Department of Cardiology, The First Hospital of Hebei Medical University, 89 Donggang Road, Shijiazhuang 050000, Hebei, China; ^2^Graduate School of Hebei Medical University, 361 Zhongshan East Road, Shijiazhuang 050000, Hebei, China

## Abstract

**Background:**

Atrial fibrillation (AF) is one of the most common arrhythmias in clinical practice. Although fat is currently considered to be a risk factor for AF and a pathogenic link between epicardial fat tissue (EFT) and AF has been speculated, there are currently few clinical studies and literature data domestically or abroad.

**Objective:**

This study conducted a meta-analysis of observational case series studies to verify the relationship between atrial fibrillation and EFT and to strengthen the predictive value of EFT in the occurrence, development, and postablative recurrence of AF.

**Methods:**

We conducted a systematic search of the literature in electronic databases until December 2021 and supplemented this through manual searches of individual studies, reviewed articles, and reference lists in conference proceedings. This study conducted a meta-analysis to compare the differences between different populations, such as healthy participants and AF patients, healthy subjects and AF subtype cases, and paroxysmal and persistent AF with AF recurrence and without AF recurrence after ablation.

**Results:**

Following the retrieval of 828 articles, only 22 articles were selected as research results. Accordingly, the meta-analysis results show that the volume of EFT in AF is greater than that in healthy subjects (MD = 39.34 ml, 95% CI = 27.11, 51.58); persistent AF is greater than paroxysmal AF (MD = 14.37 ml, 95% CI = 7.46, 21.27); and recurrence after ablation is greater than without recurrence (MD = 14.37 ml, 95% CI = 7.46, 21.27).

**Conclusion:**

The results of this study further confirm the connection between EFT and AF and that EFT has a certain predictive value for the occurrence and development of AF.

## 1. Introduction

AF is the most common clinically sustained cardiac arrhythmia and a significant contributor to cardiovascular morbidity and mortality, with increasing morbidity and prevalence worldwide. The currently estimated prevalence of AF in adults is between 2% and 4% [1]. Therefore, it is very important to find one or more predictive indicators for AF. Regarding the treatment of AF, although catheter ablation is currently the best treatment option for the control of symptomatic AF while being more effective than antiarrhythmic drug therapy to maintain sinus rhythm, there is nevertheless a high rate of postoperative recurrence of AF (approximately 30%-50%) and difficulty in predicting rhythmic outcomes after catheter ablation of AF in individual patients [2].

Previous studies have demonstrated a close link between AF and EFT, but the mechanism by which fat causes AF remains unclear. Some studies have pointed out that fat is associated with diastolic dysfunction, atrial inflammation, myocardial deposition, and atrial systolic function disorders, which may lead to atrial structural remodeling (including diffuse atrial fibrosis and dilation) and electrophysiological abnormalities (including conduction slowing and shortening of the atrial effective refractory period) [3–5]. As a special visceral fat tissue, EFT is not only anatomically close to the myocardium but can also produce a variety of cytokines with proinflammatory effects. Atrial fibrosis is a central pathophysiological feature of AF [4].

It has been suggested that EFT has an additional role in modulating different triggers, including metabolic and biochemical triggers, that contribute to the development of AF. The interaction between AF and EFT is both structural and functional, with atrial structural abnormalities, adipocyte infiltration, and atrial fibrosis predisposing myocardial tissue to arrhythmia [6]. To date, there have been systematic reviews and meta-analyses on the relationship between EFT and AF, but the research results contained in the articles are few [7, 8]. This study contains more findings and comprehensively describes the predictive value of EFT in the occurrence, development, and recurrence after ablation of AF.

## 2. Methods

### 2.1. Search Strategy

The PubMed, Cochrane Library, Embase, and CNKI databases were manually searched for relevant literature. Searches were performed using the following keywords: “atrial fibrillation, arrhythmia, heart, fat tissue, epicardial fat, and epicardial adipose.” Titles and abstracts were screened to exclude irrelevant articles. In addition, the references of all ultimately included articles were reviewed to prevent any relevant articles from being missed.

### 2.2. Qualification Criteria

There were no language restrictions on the included articles. The criteria for inclusion in the manuscript were as follows: (1) the article reported the total volume of EFT with statistical indicators, (2) the total volume of EFT was measured by CT or MRI, (3) there were healthy subjects and an AF group or AF ablation treatment group, and (4) at least one of the following major confounders was reported: age, sex, hypertension, and body mass index (BMI). Studies published as conference abstracts were considered eligible for inclusion; however, case reports and review clauses were not.

### 2.3. Assessment of Study Quality

These articles were independently assessed by two experienced clinical staff and discussed and revised in the event of disagreement. The STROBE statement was used to assess the methodological quality of the included studies, and STROBE contained 22 items with which to assess the quality of the information reported in different sections of the study, including presentation, study design and setting, statistical evaluation, results, and discussion [9]. The STROBE judgment results for all included studies were summarized on a scale between 0 and 22. In case of discrepancies, quality assessments were performed by 2 different graders and a 3rd grader. For meeting abstracts, another type of STROBE checklist was used, which contained 12 items to include when reporting observational studies published as meeting abstracts.

### 2.4. Statistical Methods

Random-effects meta-analysis was used to estimate the differences in total EFT volume among the different populations. RevMan 5.3 and Stata 15.1 were used for statistical analysis in this study, and the results were represented by forest plots. Since the included studies were all measurement data, mean difference (MD) and 95% confidence intervals (CIs) were used to evaluate whether there was a significant difference in EFT volume between different populations. At the significance level *a* = 0.1, the heterogeneity test adopted was the *X*^2^ test, *P* ≥ 0.1, with *I*^2^ ≤ 50% indicating that the heterogeneity was small and for which the fixed-effect model (FED) would be used; otherwise, the random-effect model (RED) would be used. Subgroup analysis and drawing a funnel plot were used to assess potential publication bias.

## 3. Results

**Figure 1:**
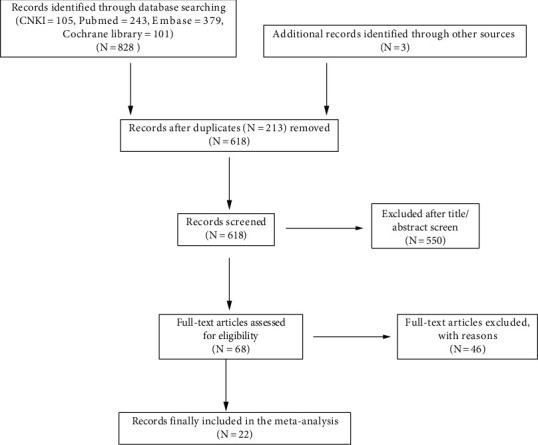
Flow chart of study selection.

A total of 831 literature search results were recorded. After 213 copies of the records were removed, 550 of the remaining 618 records were not related to the subject. After evaluating the full text of the remaining 68 studies, 46 of them were excluded. Finally, the results of 22 published studies were included in this study. Finally, 5 different meta-analyses were performed to assess the association of EFT volume with AF. Flowchart of study selection is reported in Figure 1. The characteristics of the included studies are reported in Table 1.

### 3.1. Comparison of EFT Volume in Healthy Participants and AF Cases

**Figure 2:**
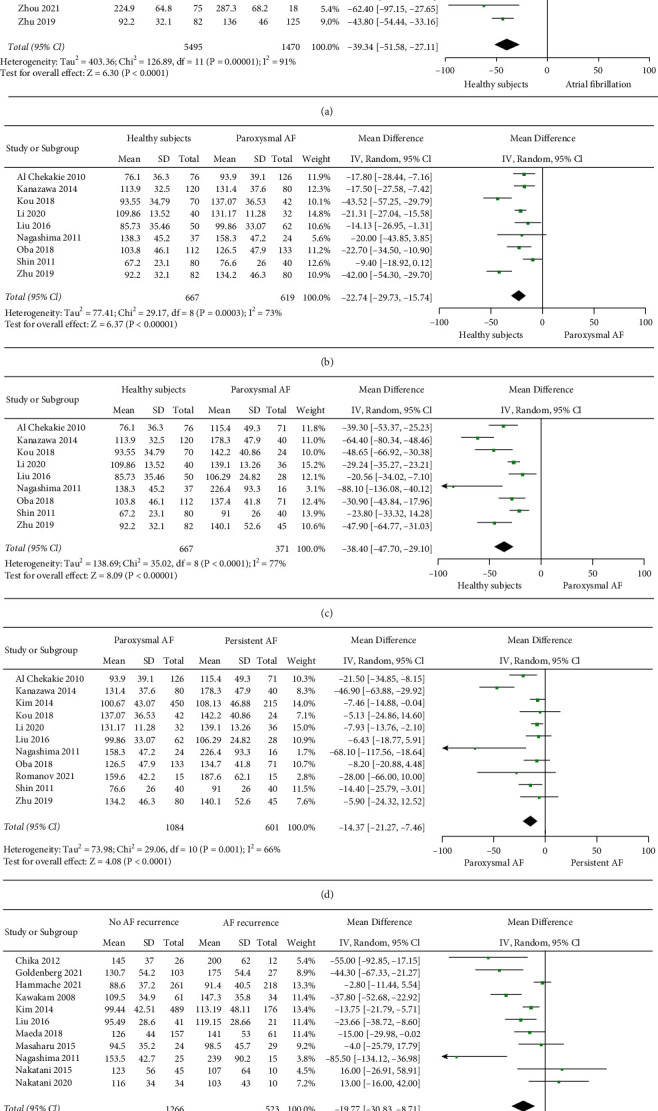
Forest map of EFT volume differences among different populations: (a) healthy participants and all AF cases; (b) healthy participants and paroxysmal atrial fibrillation; (c) healthy participants and persistent atrial fibrillation; (d) paroxysmal atrial fibrillation and persistent atrial fibrillation; and (e) recurrent and nonrecurring patients after ablation.

**Figure 3:**
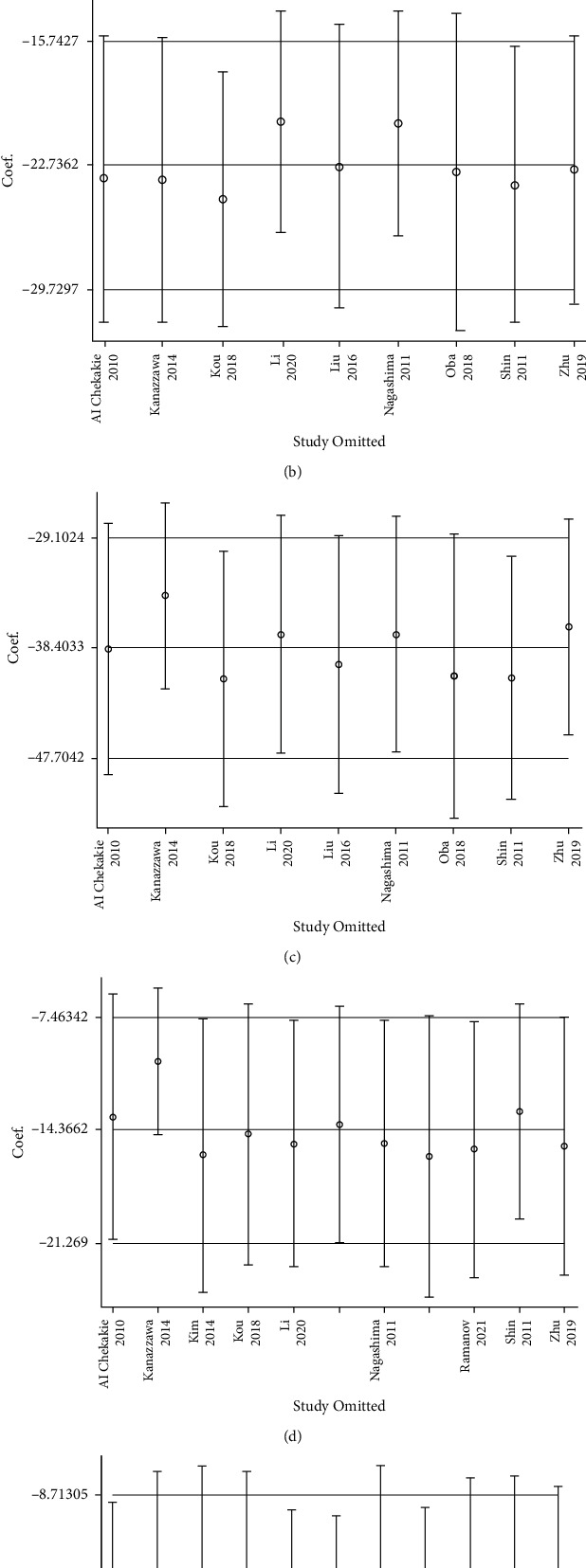
Sensitivity analysis plot of EFT volume differences among different populations: (a) healthy participants and all AF cases; (b) healthy participants and paroxysmal atrial fibrillation; (c) healthy participants and persistent atrial fibrillation; (d) paroxysmal atrial fibrillation and persistent atrial fibrillation; and (e) recurrent and nonrecurring patients after ablation.

Analysis of 5495 healthy controls and 1470 AF subjects using a random-effects model showed that the MD was −39.34 ml (95% confidence interval (CI) = −51.58, −27.11), indicating that the EFT volume was higher in AF cases. From this comparison, relative heterogeneity between studies was observed (*I*-squared = 91% *P* < 0.00001) (Figure 2(a)). Inclusion of potential confounding factors for supplementation, such as sex, incidence of type 2 diabetes, and hypertension, did not result in a reduction in supplementation heterogeneity. After excluding individual studies one by one, the combined MD values were all within the 95% CI (−51.58, −27.11), and the MD values ranged from −41.66 (95% CI = −54.33, −28.98) to −35.32 (95% CI = −41.59, −25.46). The results obtained in this study were relatively stable (Figure 3(a)).

### 3.2. Comparison of Total EFT Volume in Healthy Subjects and AF Subtype Cases

A comparison of total EFT volume between 667 patients in sinus rhythm and 619 patients with paroxysmal AF (PAF) using a random-effects model showed an MD of −22.74 ml (95% confidence interval (CI) = −29.73, −15.74). From this comparison, relative heterogeneity between studies was observed (*I*-squared = 73% *P* < 0.00001) (Figure 2(b)). After excluding individual studies one by one, the combined MD values were all within the 95% CI (−29.73, −15.74), and the MD values ranged from −24.61 ml (95% CI = −31.69, −17.54) to −20.21 ml (95% CI = −26.33, −14.08) (Figure 3(b)). When comparing healthy subjects (*n* = 667) and patients (*n* = 371) with persistent AF (PeAF), the MD was −38.40 ml (95% CI = −47.70, −29.10), and relative heterogeneity between studies was also observed (*I*-squared = 77% *P* < 0.00001) (Figure 2(c)). After excluding individual studies one by one, the combined MD values were all within the 95% CI (−47.70, −29.10), and the MD values ranged from −41.00 ml (95% CI = −51.56, −30.45) to −34.01 ml (95% CI = −41.74, −26.31) (Figure 3(c)). The above results show that the results obtained in this study were relatively stable and that the total EFT volume was significantly higher in AF.

### 3.3. Comparison of Total EFT Volume in Paroxysmal AF and Persistent AF

Notably, when comparing patients with PAF (*n* = 1084) and PeAF (*n* = 610), a significant MD of −14.37 ml (95% CI = −21.27, −7.46) was found, showing that the EFT volume was higher in PeAF cases. From this comparison, relative heterogeneity between studies was observed (*I*-squared = 66% *P* < 0.0001) (Figure 2(d)). After excluding individual studies one by one, the combined MD values were all within the 95% CI (−21.27, −7.46), and the MD values ranged from −15.94 ml (95% CI = −24.35, −7.54) to −10.16 ml (95% CI = −14.53, −5.78), showing that the results were relatively stable (Figure 3(d)).

### 3.4. Comparison of AF Recurrence and Nonrecurrence after Ablation

The comparison of total EFT volume between the two groups of patients with (*n* = 523) and without (*n* = 1266) AF recurrence after ablation using a random-effects model showed an SDM of −19.77 ml (95% CI = −30.83, −8.71), indicating that EFT volume was higher in AF recurrence patients. In addition, relative heterogeneity between studies was observed (*I*-squared = 77% *P*=0.0005) (Figure 2(e)). After excluding individual studies one by one, the combined MD values were all within the 95% CI (−30.83, −8.71), and the MD values ranged from −22.41 ml (95% CI = −34.43, −10.39) to −17.15 ml (95% CI = −27.54, −6.75), showing that the results were relatively stable (Figure 3(e)).

### 3.5. Quality Assessment and Publication Bias

**Figure 4:**
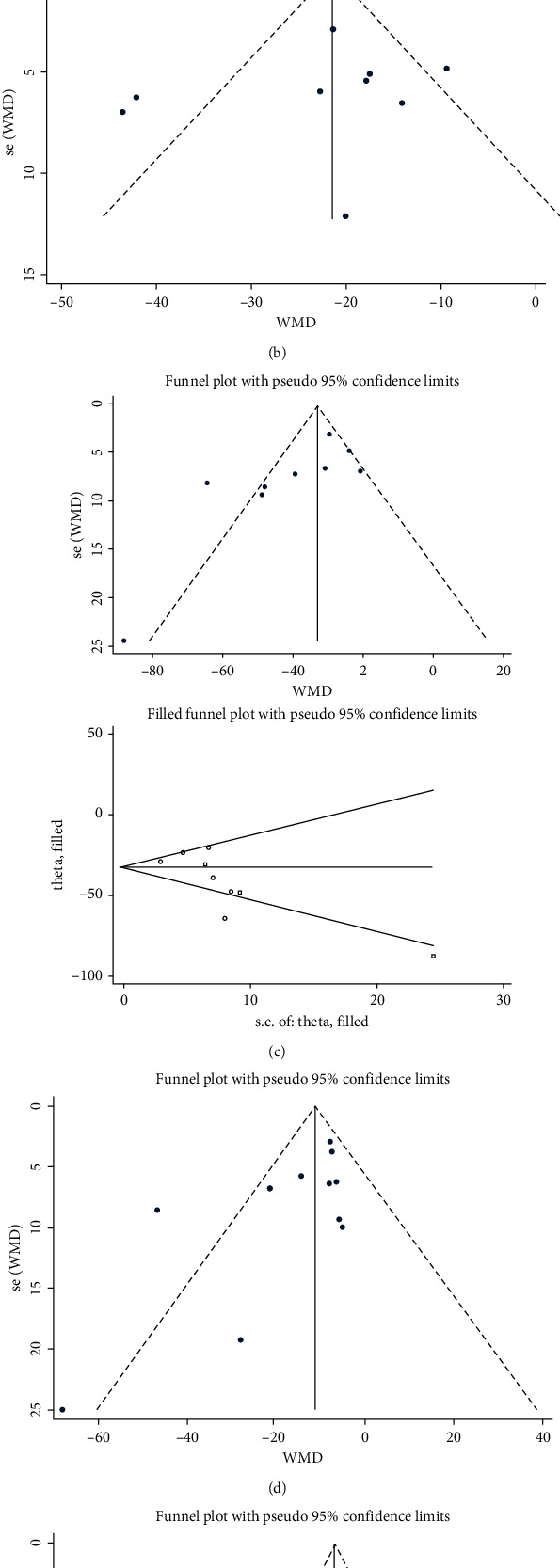
Funnel plot of EFT volume differences among different populations: (a) healthy participants and all AF cases; (b) healthy participants and paroxysmal atrial fibrillation; (c)) healthy participants and persistent atrial fibrillation; (d) paroxysmal atrial fibrillation and persistent atrial fibrillation; and (e) recurrent and nonrecurring patients after ablation.

The quality of the studies in the current meta-analysis was variable; the lowest STROBE (22 items) score was 13, and the highest was 20; the lowest STROBE (12 items) score was 4, and the highest was 5. This meta-analysis was not affected by publication bias according to the Egger test for the following comparisons: (1) the group of healthy participants and AF cases, *P*=0.283 (Figure 4(a)); (2) the group of healthy participants and paroxysmal AF cases, *P*=0.544 (Figure 4(b)); (3) the group of healthy participants and persistent AF cases, *P*=0.045 (Figure 4(c))—therefore, we carried out a cut-and-fill analysis, and the MD value and orientation were unchanged (Figure 4(c)); (4) the group of PAF and PeAF cases, *P*=0.07 (Figure 4(d)); and (5) the AF recurrence group and the AF nonrecurrence after ablation group, *P*=0.229 (Figure 4(e)).

## 4. Discussion

### 4.1. Main Findings

This study further strengthens the link between EFT volume and AF. Since different subtypes of AF exist and since AF presents differently at different stages of development, it is difficult to predict the recurrence rate of patients with AF after radiofrequency ablation. Consequently, this study conducted a meta-analysis to determine the relationship between EFT and AF. The results of this study found that patients with AF had greater EFT volume than those with sinus rhythm and that patients with persistent AF had greater EFT volume than those with paroxysmal AF. In addition, the EFT volume of patients with AF recurrence after ablation was greater than that of patients without AF recurrence. These results further indicate that EFT is related not only to the occurrence of AF but also to the severity of AF, which strengthens the value of EFT volume as an imaging indicator in clinical work. However, further well-designed studies are still needed for more accurate assessments.

### 4.2. Link between AF and EFT

In recent years, a number of risk factors and conditions associated with AF have been identified, such as coronary heart disease, hypertension, heart failure, diabetes, smoking, age, and obesity [32]. However, the exact pathophysiological mechanism of the occurrence and progression of AF is unknown and may be related to inflammation, oxidative stress, endothelial dysfunction, microvascular dysfunction, hypercoagulability, epicardial fat tissue disturbance, atrial stretch, electrophysiology, or conduction function changes [33, 34]. Among these risk factors and mechanisms, the role played by EFT has received increasing attention. Many studies have shown that EFT is closely related to the occurrence and development of AF. The presence of other cardiovascular risk factors (age, hypertension, diabetes, and obesity) known to be associated with AF did not attenuate the relationship between AF and EFT volume [35].

Regarding the pathophysiological mechanism, some studies suggest that EFT may affect the atrial stroma through multiple pathways, such as inflammatory pathways, cardiac structural remodeling, and inducing atrial fibrosis. Atrial fibrosis has become an important pathophysiological factor in AF and has been linked to AF recurrence, resistance to therapy and complications. Epicardial adipocytes in contact with cardiomyocytes can not only infiltrate the myocardium but also secrete a large number of cytokines (IL-6, IL-4, IL-1, leptin, TNF-*α*, extracellular vesicles, etc.) regulating myofibroblast and myocyte physiology [36]. These cytokines are protective in a healthy heart against inflammation and fibrosis; however, adipokines secreted by adipocytes may be converted to proinflammatory and profibrotic cytokines and are associated with the production of reactive oxygen species [37]. In addition, EFT can also promote the occurrence of AF by affecting sympathetic nerve excitability, and autonomic nervous system dysfunction may also alter the endocrine activity of adipocytes in a feedback response [38]. Some studies have demonstrated that epicardial adipocytes are sensitive to catecholamine stimulation, which can activate the secretion of cytokines from these cells to the neighboring tissue. Atrial fibrosis is tightly associated with EFT, but the exact mechanism by which atrial myocytes and fibroblasts are associated with EFT is not fully understood [39].

### 4.3. Link between AF Severity and EFT

The severity of AF can be reflected by the duration of atrial fibrillation and the type of AF. We usually think that PeAF is often more harmful than PAF because PeAF is more likely to cause more serious clinical problems, such as heart failure. The main pathological mechanism of AF is atrial fibrosis, and PeAF is usually considered more severe fibrosis than PAF. As mentioned above, EFT can promote the progression of atrial fibrosis by secreting proinflammatory factors and direct infiltration. Previous studies have also shown that the volume of EFT and AF burden have a dose-dependent relationship, which is consistent with our finding that patients with PeAF have greater EFT volume than patients with PAF [21]. Therefore, we believe that EFT volume can reflect the severity of AF to some extent.

### 4.4. Link between Recurrence of AF and EFT

Currently, catheter ablation is an effective therapy to maintain sinus rhythm, but it is unfortunate that a long history of AF and severe atrial fibrosis can increase the probability of AF recurrence. As mentioned above, EFT promotes the occurrence of atrial fibrosis and increases AF burden through direct infiltration, secretion of adipocytokines, induction of inflammatory responses, etc. In addition, previous research has shown that resistin (an adipocytokine) and high-sensitivity c-reactive protein (an indicator of inflammation) are higher in patients with AF recurrence after catheter ablation [40, 41]. Our study shows that patients with AF recurrence after catheter ablation have a greater EFT volume. Therefore, we believe that EFT volume has some predictive value for the recurrence of AF after catheter ablation.

### 4.5. Recent Research and Treatment Progress

EFT is responsive to glucagon-like peptide 1 receptor agonists (GLP1A) and sodium glucose cotransporter 2 inhibitors (SGLT2i). As recently demonstrated, GLP-1A and SGLT2i provide weight loss and cardiovascular protection beyond diabetes control [42]. In addition, classic RAS blockers, such as angiotensin converting enzyme inhibitors (ACE-Is) and angiotensin receptor inhibitors (ARBs), may prevent AF by affecting the accumulation of EFT, especially in patients with heart failure and known left heart failure [43].

### 4.6. Limitations

Given the heterogeneity, this study used a random-effects model in the EFT meta-analysis. Then, we conducted a subgroup analysis on the three confounding factors of sex, hypertension, and diabetes, in which hypertension and diabetes did not markedly reduce the heterogeneity. The number of studies, no language restrictions on the included articles, BMI, age, and errors in manual measurements of EFT volume may all be sources of heterogeneity. However, most of the articles did not explicitly give detailed data related to BMI and age, so further analysis could not be carried out.

## 5. Conclusion

The EFT volume is associated with AF and has some predictive value in the occurrence, development, and recurrence of AF. More research is necessary to better understand the link between AF and epicardial fat tissue.

## Figures and Tables

**Table 1:** Characteristics of the included studies.

Reference year	Country	Healthy subjects (*n*) EFTV (*M* ± SD)	All AF subjects (*n*) EFTV (*M* ± SD)	PAF subjects (*n*) EFTV (*M* ± SD)	PeAF subjects (*n*) EFTV (*M* ± SD)	AF recurrence (*n*) EFTV (*M* ± SD)	No AF recurrence (*n*) EFTV (*M* ± SD)	Imaging system	Quality score
Al Chekakie et al. [10] 2010	USA	76.10 ± 36.30 *n* = 76	101.60 ± 44.10 *n* = 197	93.90 ± 39.10 *n* = 126	115.40 ± 49.30 *n* = 71	N/A	N/A	CT	15/22
Goldenberg et al. [11] 2021	Israel	55.90 ± 17.70 *n* = 50	140.30 ± 58.10 *n* = 130	N/A	N/A	130.70 ± 54.20 *n* = 103	175.00 ± 54.40 *n* = 27	CT	20/22
Greif et al. [12] 2013	Germany	255.70 ± 127.19 *n* = 934	284.81 ± 139.21 *n* = 354	N/A	N/A	N/A	N/A	CT	16/22
Hammache et al. [13] 2021	France	N/A	N/A	N/A	N/A	88.60 ± 37.20 *n* = 128	91.40 ± 40.50 *n* = 261	CT	20/22
Kanazawa et al. [14] 2014	Japan	113.90 ± 32.50 *n* = 120	148.80 ± 46.10 *n* = 120	131.40 ± 37.60 *n* = 80	178.30 ± 47.90 *n* = 40	N/A	N/A	CT	14/22
Kim et al. [15] 2014	Korea	N/A	N/A	100.67 ± 43.07 *n* = 450	108.13 ± 46.88 *n* = 215	99.44 ± 42.51 *n* = 489	113.19 ± 48.11 *n* = 176	CT	17/22
Maeda et al. [16] 2018	Germany	N/A	N/A	N/A	N/A	126.00 ± 44.00 *n* = 157	141.00 ± 53.00 *n* = 61	CT	13/22
Mahabadi et al. [17] 2014	Japan	92.70 ± 46.10 *n* = 3809	147.10 ± 64.40 *n* = 46	N/A	N/A	N/A	N/A	CT	19/22
Nagashima et al. [18] 2011	Japan	138.30 ± 45.20 *n* = 37	185.60 ± 76.10 *n* = 40	158.3 ± 47.2 *n* = 24	226.40 ± 93.30 *n* = 16	153.50 ± 42.70 *n* = 25	239.00 ± 90.20 *n* = 15	CT	18/22
Nakatani et al. [19] 2015	Japan	N/A	N/A	N/A	N/A	123.00 ± 56.00 *n* = 45	107.0 ± 64.00 *n* = 10	CT	17/22
Nakatani et al. [20] 2020	Japan	N/A	N/A	N/A	N/A	116.00 ± 34.00 *n* = 34	103.00 ± 43.00 *n* = 10	CT	18/22
Oba et al. [21] 2018	Japan	103.80 ± 46.10 *n* = 112	129.30 ± 46.00 *n* = 204	126.5 ± 47.90 *n* = 133	134.70 ± 41.80 *n* = 71	N/A	N/A	CT	16/22
Romanov et al. [22] 2021	Japan	N/A	N/A	159.60 ± 42.20 *n* = 15	187.60 ± 62.10 *n* = 15	N/A	N/A	CT	15/22
Shin et al. [23] 2011	Korea	67.20 ± 23.10 *n* = 80	83.80 ± 26.80 *n* = 80	76.60 ± 26.00 *n* = 40	91.00 ± 26.00 *n* = 40	N/A	N/A	CT	18/22
Zhou et al. [24] 2021	China	224.90 ± 64.80 *n* = 75	287.3 ± 68.2 *n* = 18	NA	NA	N/A	N/A	MRI	19/22
Zhu et al. [25] 2019	China	92.20 ± 32.10 *n* = 82	136.00 ± 46.00 *n* = 125	134.20 ± 46.30 *n* = 80	140.1 ± 52.6 *n* = 45	N/A	N/A	CT	14/22
Chika et al. [26] 2012	Japan	N/A	N/A	N/A	N/A	145.00 ± 37.00 *n* = 26	200.00 ± 62.00 *n* = 112	CT	5/12
Kawakam et al. [27] 2008	Japan	N/A	N/A	N/A	N/A	109.50 ± 34.90 *n* = 61	147.30 ± 35.80 *n* = 34	CT	4/12
Masaharu et al. [28] 2015	USA	N/A	N/A	N/A	N/A	94.50 ± 35.20 *n* = 24	98.50 ± 45.70 *n* = 29	CT	21/22
Liu et al. [29] 2016	China	85.73 ± 35.46 *n* = 50	101.86 ± 30.74 *n* = 90	99.86 ± 33.07 *n* = 62	106.29 ± 24.82 *n* = 28	95.49 ± 28.60 *n* = 41	119.15 ± 28.66 *n* = 21	CT	18/22
Kou et al. [30] 2018	China	93.55 ± 34.79 *n* = 70	138.94 ± 37.93 *n* = 66	137.07 ± 36.53 *n* = 42	142.20 ± 40.86 *n* = 24	N/A	N/A	CT	19/22
Li et al. [31] 2020	China	109.86 ± 13.52 *n* = 40	NA	131.17 ± 11.28 *n* = 32	139.10 ± 13.26 *n* = 36	N/A	N/A	CT	17/22

## Data Availability

The data are available from the first author upon request.

## References

[1] Hindricks G., Potpara T., Dagres N. (2021). 2020 ESC guidelines for the diagnosis and management of AF developed in collaboration with the European association for cardio-thoracic surgery (EACTS): the task force for the diagnosis and management of AF of the European society of cardiology (ESC) developed with the special contribution of the European heart rhythm association (EHRA) of the ESC. *European Heart Journal*.

[2] Kirchhof P., Benussi S., Kotecha D. (2016). 2016 ESC Guidelines for the management of AF developed in collaboration with EACTS. *European Heart Journal*.

[3] Moreira L. M., Takawale A., Hulsurkar M. (2020). Paracrine signalling by cardiac calcitonin controls atrial fibrogenesis and arrhythmia. *Nature*.

[4] Wong C. X., Ganesan A. N., Selvanayagam J. B. (2017). Epicardial fat and atrial fibrillation: current evidence, potential mechanisms, clinical implications, and future directions. *European Heart Journal*.

[5] Goudis C. A., Vasileiadis I. E., Liu T. (2018). Epicardial adipose tissue and atrial fibrillation: pathophysiological mechanisms, clinical implications, and potential therapies. *Current Medical Research and Opinion*.

[6] Schotten U., Verheule S., Kirchhof P., Goette A. (2011). Pathophysiological mechanisms of atrial fibrillation: a translational appraisal. *Physiological Reviews*.

[7] Sepehri Shamloo A., Dagres N., Dinov B. (2019). Is epicardial fat tissue associated with atrial fibrillation recurrence after ablation? a systematic review and meta-analysis. *IJC Heart & Vasculature*.

[8] Gaeta M., Bandera F., Tassinari F. (2017). Is epicardial fat depot associated with atrial fibrillation? a systematic review and meta-analysis. *EP Europace*.

[9] Von Elm E., Altman D. G., Egger M., Pocock S. J., Gotzsche P. C., Vandenbroucke J. P. (2014). The strengthening the reporting of observational studies in epidemiology (STROBE) statement: guidelines for reporting observational studies. *International Journal of Surgery*.

[10] Al Chekakie M. O., Welles C. C., Metoyer R. (2010). Pericardial fat is independently associated with human atrial fibrillation. *Journal of the American College of Cardiology*.

[11] Goldenberg G. R., Hamdan A., Barsheshet A. (2021). Epicardial fat and the risk of atrial tachy-arrhythmia recurrence post pulmonary vein isolation: a computed tomography study. *International Journal of Cardiovascular Imaging*.

[12] Greif M., von Ziegler F., Wakili R. (2013). Increased pericardial adipose tissue is correlated with atrial fibrillation and left atrial dilatation. *Clinical Research in Cardiology*.

[13] Hammache N., Pegorer-Sfes H., Benali K. (2021). Is there an association between epicardial adipose tissue and outcomes after paroxysmal atrial fibrillation catheter ablation?. *Journal of Clinical Medicine*.

[14] Kanazawa H., Yamabe H., Enomoto K. (2014). Importance of pericardial fat in the formation of complex fractionated atrial electrogram region in atrial fibrillation. *International Journal of Cardiology*.

[15] Kim T. H., Park J., Park J. K. (2014). Pericardial fat volume is associated with clinical recurrence after catheter ablation for persistent atrial fibrillation, but not paroxysmal atrial fibrillation: an analysis of over 600-patients. *International Journal of Cardiology*.

[16] Maeda M., Oba K., Yamaguchi S. (2018). Usefulness of epicardial adipose tissue volume to predict recurrent atrial fibrillation after radiofrequency catheter ablation. *American Journal of Cardiology*.

[17] Mahabadi A. A., Lehmann N., Kälsch H. (2014). Association of epicardial adipose tissue and left atrial size on non-contrast CT with atrial fibrillation: the Heinz Nixdorf Recall Study. *European Heart Journal - Cardiovascular Imaging*.

[18] Nagashima K., Okumura Y., Watanabe I. (2011). Association between epicardial adipose tissue volumes on 3-dimensional reconstructed CT images and recurrence of atrial fibrillation after catheter ablation. *Circulation Journal*.

[19] Nakatani Y., Kumagai K., Minami K., Nakano M., Inoue H., Oshima S. (2015). Location of epicardial adipose tissue affects the efficacy of a combined dominant frequency and complex fractionated atrial electrogram ablation of atrial fibrillation. *Heart Rhythm*.

[20] Nakatani Y., Sakamoto T., Yamaguchi Y., Tsujino Y., Kinugawa K. (2020). Epicardial adipose tissue affects the efficacy of left atrial posterior wall isolation for persistent atrial fibrillation. *Journal of Arrhythmia*.

[21] Oba K., Maeda M., Maimaituxun G. (2018). Effect of the epicardial adipose tissue volume on the prevalence of paroxysmal and persistent atrial fibrillation. *Circulation Journal*.

[22] Romanov A., Minin S., Nikitin N. (2021). The relationship between global cardiac and regional left atrial sympathetic innervation and epicardial fat in patients with atrial fibrillation. *Annals of Nuclear Medicine*.

[23] Shin S. Y., Yong H. S., Lim H. E. (2011). Total and interatrial epicardial adipose tissues are independently associated with left atrial remodeling in patients with atrial fibrillation. *Journal of Cardiovascular Electrophysiology*.

[24] Zhou Y., Yu M., Cui J. (2021). The predictive value of epicardial adipose tissue volume assessed by cardiac magnetic resonance for atrial fibrillation in patients with hypertrophic obstructive cardiomyopathy. *International Journal of Cardiovascular Imaging*.

[25] Zhu Y. M., Xu H. X., Lu Q., Huang Y. H., Jing H. M., Wu X. (2019). [Correlation between multi-slice spiral CT determined epicardial adipose tissue volume and atrial fibrillation]. *Zhonghua Xinxueguanbing Zazhi*.

[26] Murakami C., Nagai T., Akira F. (2012). Total epicardial fat volume is associated with early recurrence of atrial fifibrillation after catheter ablation. *Journal of the American College of Cardiology*.

[27] Kawakami H., Satomi K., Nakajima I. (2013). Total epicardial adipose tissue volume is associated with outcome of pulmonary vein isolation for atrial fifibrillation. *Europace*.

[28] Masuda M., Mizuno H., Enchi Y. (2015). Abundant epicardial adipose tissue surrounding the left atrium predicts early rather than late recurrence of atrial fibrillation after catheter ablation. *Journal of Interventional Cardiac Electrophysiology*.

[29] Liu Y., Liu S., Qian X. I. N. (2016). CorrelatiOn of epicardjal adipose tissue Volume with atriaI fibrillation and its recurrence. *Chinese Journal of Geriatric Heart Brain and Vessel Diseases*.

[30] Chen-guang K. O. U., Cai-ying L. I., Fang-ying J. I. A. (2018). Correlation study between epicardial fat volume detected by 256 slice spiral CT and atrial fibrillation. *Chinese Circulation Joural*.

[31] Yi-fan L., Guo Z.-p. (2020). Correlation between epicardial adipose tissue volume and elderly atrial fibrillation. *Chinese Journal of Gerontology*.

[32] Schnabel R. B., Yin X., Gona P. (2015). 50 year trends in atrial fibrillation prevalence, incidence, risk factors, and mortality in the Framingham Heart Study: a cohort study. *Lancet*.

[33] Wijesurendra R. S., Casadei B. (2019). Mechanisms of atrial fibrillation. *Heart*.

[34] Nattel S., Heijman J., Zhou L., Dobrev D. (2020). Molecular basis of atrial fibrillation pathophysiology and therapy: a translational perspective. *Circulation Research*.

[35] Couselo-Seijas M., Rodríguez-Mañero M., González-Juanatey J. R., Eiras S. (2021). Updates on epicardial adipose tissue mechanisms on atrial fibrillation. *Obesity Reviews: An Official Journal of the International Association for the Study of Obesity*.

[36] Shaihov-Teper O., Ram E., Ballan N. (2021). Extracellular vesicles from epicardial fat facilitate atrial fibrillation. *Circulation*.

[37] Krishnan A., Chilton E., Raman J. (2021). Are interactions between epicardial adipose tissue, cardiac fibroblasts and cardiac myocytes instrumental in atrial fibrosis and atrial fibrillation?. *Cells*.

[38] Balcioğlu A. S., Çiçek D., Akinci S. (2015). Arrhythmogenic evidence for epicardial adipose tissue: heart rate variability and turbulence are influenced by epicardial fat thickness. *Pacing and Clinical Electrophysiology*.

[39] Nattel S. (2017). Molecular and cellular mechanisms of atrial fibrosis in atrial fibrillation. *Journal of the American College of Cardiology: Clinical Electrophysiology*.

[40] Malouf J. F., Kanagala R., Al Atawi F. O. (2005). High sensitivity C-reactive protein: a novel predictor for recurrence of atrial fibrillation after successful cardioversion. *Journal of the American College of Cardiology*.

[41] Pokushalov E., Kozlov B., Romanov A. (2014). Botulinum toxin injection in epicardial fat pads can prevent recurrences of atrial fibrillation after cardiac surgery: results of a randomized pilot study. *Journal of the American College of Cardiology*.

[42] Iacobellis G., Baroni M. G. (2022). Cardiovascular risk reduction throughout GLP-1 receptor agonist and SGLT2 inhibitor modulation of epicardial fat. *Journal of Endocrinological Investigation*.

[43] Mascolo A., Urbanek K., De Angelis A. (2020). Angiotensin II and angiotensin 1–7: which is their role in atrial fibrillation?. *Heart Failure Reviews*.

